# Insulin resistance and associated factors: a cross-sectional study of bank employees

**DOI:** 10.6061/clinics/2017(04)06

**Published:** 2017-04

**Authors:** Luciane Bresciani Salaroli, Monica Cattafesta, Maria del Carmen Bisi Molina, Eliana Zandonade, Nazaré Souza Bissoli

**Affiliations:** I Programa de Pós Graduação em Saúde Coletiva, Programa de Pós Graduação em Nutrição e Saúde, Departamento de Educação Integrada em Saúde, Universidade Federal do Espírito Santo, Vitória, ES, BR; II Programa de Pós Graduação em Nutrição e Saúde, Universidade Federal do Espírito Santo, Vitória, ES, BR; III Programa de Pós Graduação em Saúde Coletiva, Universidade Federal do Espírito Santo, Vitória, ES, BR; IV Programa de Pós Graduação em Ciências Fisiológicas, Universidade Federal do Espírito Santo, Vitória, ES, BR

**Keywords:** Insulin Resistance, Bank Employees, Metabolic Syndrome

## Abstract

**OBJECTIVE:**

Insulin resistance is characterized by the failure of target cells to respond to normal levels of circulating insulin, and this condition is related to cardiovascular disease. This study sought to evaluate the prevalence of insulin resistance and its association with markers of metabolic abnormalities and metabolic syndrome in bank employees.

**METHODS:**

A cross-sectional study was performed on 498 working men and women aged ≥20 years old. The Homeostasis Model Assessment (HOMA-IR) was used to determine the presence of insulin resistance based on cut-off values of ≤2.71 for normal insulin levels and >2.71 for insulin resistance, as established for the adult Brazilian population.

**RESULTS:**

It was observed that the 52 (10.4%) overweight individuals with insulin resistance were 4.97 times (95%CI 1.31-18.83) more likely to have high HOMA-IR values than the normal-weight participants; among those who were obese, the likelihood increased to 17.87 (95%CI 4.36-73.21). Individuals with large waist circumferences were 3.27 times (95%CI 1.03-10.38) more likely to develop insulin resistance than those who were within normal parameters. The HOMA-IR values differed between subjects with and without metabolic syndrome, with values of 2.83±2.5 and 1.10±0.81 (*p*=0.001), respectively. The levels of insulin, ultrasensitive C-reactive protein and uric acid were also associated with insulin resistance.

**CONCLUSION:**

The prevalence of insulin resistance among bank employees is high, and insulin resistance is associated with and serves as a marker of metabolic syndrome. Cardiovascular disease and metabolic syndrome-associated metabolic abnormalities were observed, and insulin resistance may be a risk factor in this group of professionals.

## INTRODUCTION

Insulin resistance (IR) is characterized by the failure of target cells, including adipose, pancreas, skeletal muscle and liver tissues, to respond to normal levels of circulating insulin. IR occurs because of genetic, nutritional and metabolic disorders 1 and results in compensatory hyperinsulinaemia in an attempt to obtain a proper physiological response 2.

The metabolic disorders that occur in IR are involved in the pathogenesis of type 2 diabetes mellitus (type 2 DM), and when associated with dyslipidaemia, obesity and hypertension (AH), these conditions constitute metabolic syndrome (MS) 3. MS is characterized by a complex network of nutritional and metabolic disorders, including inflammation, oxidative stress, vitamin D deficiency, anaemia and high levels of C reactive protein (CRP) 3,4.

MS has been the subject of increasing concern worldwide because it is related to an increased risk of cardiovascular disease (CVD) 3,5, which may be linked to IR because of increased visceral fat deposition 6.

Classically, the gold standard for measuring IR is the euglycaemic hyperinsulinaemic clamp; however, its use in large populations is limited because it is invasive, costly and complex 7. Accordingly, simpler methods can be used to identify individuals with IR. In 1985, Mattheus et al. 8 published the Homeostasis Model Assessment (HOMA), which shows a strong correlation with clamp results 9 and has been validated for epidemiological studies by several authors; however, this method is still not recommended for use in clinical practice 7.

As IR is a multifactorial condition that includes an increase in inflammatory markers, changes in lipid metabolism and changes in the intestinal microbiota, which are all interconnected to different degrees, early identification of this metabolic change presents the possibility of disease prevention and improved quality of life 10.

Bank employees are a group of workers with high occupational stress 11, who are at increased risk of CVD 12-14 and present with a high occupational illness risk due to their extremely stressful daily working life and changes in the labour market 12,15. Moreover, this group represents a variety of population classes and workers. In large Brazilian cities, bank employees constitute a large category of young adults and middle-aged middle-class individuals in the service sector, and their jobs are threatened by rapid advances in automation.

This study aimed to assess the prevalence of IR and its association with markers of metabolic abnormalities and MS in bank employees.

## METHODS

### Ethics Statement

This study was approved by the Research Ethics Committee (number 059/08) of the Centre for Health Sciences, Federal University of Espírito Santo, and written informed consent was obtained from all participants.

### Study population

We conducted an observational cross-sectional study of employees, aged 20 to 64 years old, at a state-owned banking network located in southeastern Brazil. Data were collected from August 2008 to August 2009.

The sample size was calculated to estimate the prevalence of MS in a population of 1,410 bank employees. We used simple random sampling for a prevalence of 20% with a 3% error and a significance level of 95%. The 1.5 design effect was considered to offset the correlations among individuals at the same agency. Quotas were established for the type of work (general direction and agency), sex and age. Therefore, the minimum sample size was 461 bank employees. Because of a possible low response rate, 525 bank employees were invited to participate. Data collection was performed at the workplace, and the employee was relieved of his or her duties during data collection. Socioeconomic status was determined according to the Brazilian Economic Classification. Ethnicity was determined by self-classification as black, brown, white, yellow or indigenous, according to the Brazilian Institute of Geography and Statistics ethnicity categories.

## METHODS

Systolic blood pressure (SBP) and diastolic blood pressure (DBP) were measured three times during the interview for each participant, and the first measurement was discarded. Blood pressure was measured an additional time if the difference between readings was higher than 4 mmHg. For the blood pressure measurement, the OMRON 742 digital® (*OMRON Healthcare Inc.*, Shanghai, People's Republic of China), which was calibrated and validated by the National Institute of Metrology, Quality and Technology (Inmetro), was used. The individual's blood pressure levels were measured according to the criteria of the VII Joint National Committee 16. Clamps suitable for obese patients were used when needed.

All anthropometric measurements were taken by trained researchers. Body weight was obtained using a Tanita® electronic scale that was accurate to 0.1 kg (TANITA Corporation, Arlington Heights, Illinois, USA), and the individual was asked to be weighed with an empty bladder and wearing only underwear. Height was measured in metres using the Sanny® stadiometer, which was accurate to 0.1 cm (American Medical do Brasil, Ltd., São Bernardo do Campo, Brazil), while the subjects were barefoot and standing with their arms along their bodies and eyes fixed on a spot on the horizon. Body mass index was calculated (BMI=weight/height^2^) as recommended by the World Health Organization 17 to assess nutritional status. The following cut-offs were used to classify individuals according to BMI (kg/m^2^): underweight, BMI<18; normal, ≥18.5 and <25; pre-obese, ≥25 and <30; and obese ≥30. The types of obesity, including grades I to III, were pooled in the analysis so that the group could be more representative. Waist measurements were obtained using a metal tape measure. To measure the waist circumference (WC), the tape measure was placed at the midpoint between the last rib and iliac crest. WC measurements were also classified according to the WHO 17.

Biochemical tests were performed in the reference laboratory using commercial kits to measure the levels of glucose, total cholesterol (TC), high-density lipoprotein cholesterol (HDL-c), low-density lipoprotein cholesterol (LDL-c), very-low-density lipoprotein cholesterol (VLDL-c), triglycerides, uric acid, ultrasensitive CRP (us-CRP) and insulin. The insulin levels were determined using chemiluminescence with a COBAS E601 machine (Roche Diagnostics International Ltd, Rotkreuz, Switzerland). The concentration of LDL-c was calculated using the Friedewald formula 18.

The presence of MS was classified according to the National Cholesterol Education Program's Adult Treatment Panel III 19, as previously described in a previous study by our group 14.

### Definition of Insulin resistance

IR quantification was assessed using the HOMA-IR index calculated according to the formula developed by Matthews et al. 17: HOMA-IR=IF (U / mL) x FG (mmol / L) /22.5, where IF corresponds to insulin fasting and FG to fasting glucose. The cut-off used was proposed by Geloneze et al. (2009) 20 as a reference for the adult Brazilian population; IR values ≤2.71 are considered normal and free of resistance, while insulin resistance is defined as IR values >2.71.

### Statistical analyses

Statistical analyses were conducted using SPSS for Windows, version 15.0 (SPSS, Inc., Chicago, USA). The mean values were compared using the Student's t-test for independent samples. When the normality was not verified by the Kolmogorov-Smirnov test, we performed the nonparametric Mann-Whitney test. To analyse differences in proportions, we used the chi-square test (X^2^). The level of significance for all tests was set at 5%. After the bivariate analysis, logistic regression analysis was performed using the presence of MS as a dependent variable, according to the NCEP-ATP III. The crude and adjusted odds ratios (ORs) of the variables that entered the logistic regression model were calculated. All variables with *p*<0.20 in the bivariate analysis were included in the regression model. In the final model, only the variables with *p*<0.05 are shown.

## RESULTS

Of the subjects in this study (n=498), 52 (10.4%) presented with IR according to the HOMA-IR index. Of these, most were in the 41-50-year age group, belonged to classes B and C, completed or were enrolled in third grade, were white and were married.

Table 1 shows the workplace characteristics, self-perceived health status, BMI, WC and blood pressure (BP) in relation to IR. There was a significant difference in the relationship between the participant's perceptions of the variable and actual health status (*p*=0.028), BMI (*p*=0.001), WC (*p*=0.001) and BP (*p*=0.001).

Biochemical, anthropometric and haemodynamic markers in individuals with and without IR are shown in Table 2. In the study population, higher mean values for glucose, fasting insulin, total cholesterol, triglycerides and uric acid and lower levels of HDL-c were observed in individuals diagnosed with IR. The mean levels of us-CRP were significantly higher in subjects with IR. Anthropometric indicators showed a similar behaviour for both analysed criteria; therefore, the average weight values, WC and BMI were higher among IR subjects. Haemodynamic indicators were also associated with higher average SBP and DBP levels in the group with a HOMA-IR index above the cut-off point.

Table 3 shows the crude and adjusted OR values of the variables in the logistic regression model. The overweight individuals showed a 4.97-fold (95%CI 1.31-18.83) higher risk of having a higher-than-normal HOMA-IR; among those who were obese, the risk increased to 17.87 times higher (95%CI 4.36-73.21). Furthermore, the subjects with a large WC were identified as 3.27-fold (95%CI 1:03 to 10:38) more likely to develop IR than those with WCs within normal parameters.

Regarding the relationship between the HOMA-IR value and the presence or absence of conditions related to MS among bank employees, according to the NCEP-ATP III criteria, we observed values of 83±2.5 for subjects with MS and 1.10±0.81 (p=0.001) for those without MS. The index was significantly higher among subjects with low HDL-c (1.70±1.86 mg/dL in the presence of IR and 1.22±1.06 mg/dL in the absence of IR; p=0.005), elevated blood pressure (1.69±1.77 mmHg in the presence of IR and 1.14±0.96 mmHg in the absence of IR; p=0.001), abdominal obesity (2.43±2.13 cm in the presence of IR and 1.06±0.88 cm in the absence of IR; p=0.001), high triglycerides (2.07±1.89 mg/dL in the presence of IR and 1.18±1.17 mg/dL in the absence of IR; p=0.001) and increased glucose levels (3.33±2.92 mg/dL in the presence of IR and 1.30±1.24 mg/dL in the absence of IR; p=0.001) for both measurement systems.

**Figure 1:**
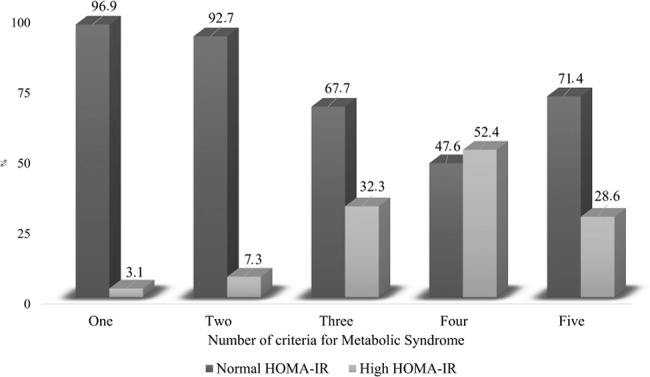
The presence of IR (HOMA-IR) according to the number of components of metabolic syndrome present in bank employees, Vitória/ES-Brazil.

Figure 1 shows the increasing trend of the HOMA-IR index with the aggregation of MS components. This trend was most intense in the presence of four components of the syndrome.

## DISCUSSION

IR is closely related to cardiovascular risk factors 21. In this context, the early identification of this metabolic change allows for disease prevention and improved quality of life 2.

In recent decades, there have been profound changes in work processes, particularly at banks, which were the subject of this study. Among these changes, corporations, mergers, privatization and outsourcing have led to a sharp reduction in the market for banking, which has led to job elimination, agency overlap, management restructuring, job mergers, and the intensive use of information technology 22. Instability and unpredictability have made jobs, especially those in the state-owned financial institutions that were once believed to provide employment for life, acquire a transitory nature as portrayed by Codo (2004) 23. Professionals in this type of work environment experience a syndrome caused by empty and meaningless work.

Therefore, the organizational changes in the banking field have resulted in the accumulation of physical and cognitive overload, unemployment, precariousness in labour relations, and illness. These factors have led to a substantial increase in the risk of acute and chronic disorders of the cardiovascular system in this population, including IR, DM, MS and other cardiovascular diseases 24.

Regarding the presence of these risk factors in the study population, IR was present in 52 (10.4%) individuals, a proportion lower than that identified in rural populations 25. Studies of IR in workers are scarce in the literature, although a population-based study conducted in Spain identified higher proportions of IR (29.9%) in patients with a mean age of 42 years 26.

The present study showed an association between BMI and WC with IR; there was also a high number of bank employees who were overweight or obese. Obesity is consistently associated with a set of metabolic diseases, including hypertension, atherosclerosis, dyslipidaemia, and type 2 DM, which commonly occur with MS 1,2,14,27,28. Furthermore, MS is characterized by hyperinsulinaemia and different IR intensities, which explains the relationship between various abnormalities and obesity 2,27. A study of the distribution of HOMA-IR values among different BMI categories for the Brazilian population showed that overweight and obese individuals had significantly higher HOMA-IR values than people with a normal weight 27, which was also demonstrated in this study.

Both weight gain and the distribution of body fat seem to greatly influence the abnormalities associated with obesity, IR and the glucose and lipid profiles; in particular, free fatty acids and their metabolites are more commonly detected in individuals who have central obesity 6. In the current study, over 50% of the individuals presented with a WC outside the normal range; in addition, individuals with a high WC and BMI showed a higher risk of developing IR compared to those with parameters within the normal range. Vasques et al. (2009) 2, in an evaluation of the ability of anthropometric indicators and body composition to identify IR, showed that WC demonstrated the best identification ability. Changes in lipid metabolism in individuals with IR are primarily triggered by excess circulating fatty acids derived from visceral adipose tissue. The insulin sensitivity of muscle tissue is also reduced by the excessive level of fatty acids due to the inhibition of glucose uptake, both from the mobilization of body fat and the presence of dyslipidaemia (low HDL-c and TC, LDL-c, VLDL-c and high triglycerides). Furthermore, hyperglycaemia and excess fatty acids can result in hyperinsulinaemia 10,29.

In addition to the excess body fat identified in the present study, there was an association between blood pressure and IR. Insulin promotes renal sodium reabsorption, and hyperinsulinaemia is expected to exacerbate that action. In the IR state, this retention effect is maintained in the kidneys, and insulin sensitivity of the proximal tubular cells is preserved. Comparisons of subjects with and without MS have shown that patients with MS have significantly increased proximal sodium reabsorption 30. The Westernized diet, which is rich in fat and salt, seems to mainly contribute to the IR process because urinary sodium excretion, which indicates the dietary consumption of sodium, is increased in MS subjects 31.

When comparing subjects with and without MS, which does not require a direct measurement of IR, the HOMA-IR values were significantly higher among individuals with MS, which has also been reported by other authors 32,33. It should be noted that this study found an association between the components of the modified MS and HOMA-IR. The results of other studies of the general population indicate that the number of individuals with a high HOMA-IR increases with the evolution of the five components of MS 34. A population study assessing workers who performed manual labour and those who did not perform this type of work showed a relationship between IR and a higher prevalence of MS in the latter type of workers 35, which demonstrates that work activities may influence the results of metabolic studies.

In addition to the diagnostic criteria for MS, other metabolic biomarkers that were altered in the study population, such as uric acid and us-CRP, were associated with IR and MS. Similar results were previously found for uric acid 33,36 and us-CRP 37 in the general population and among workers 38.

Uric acid and high us-CRP are risk factors for the onset of type 2 DM and obesity 39, and high levels of these factors can identify individuals with an inflammatory condition 38.

Self-reported health conditions are not merely impressions related to actual health, and studies in this field in Brazil have verified the high reliability and validity of such reports 40. It should be noted that 75% (n=39) of the subjects presenting with IR reported a positive health status. This finding differs from that of the study by Peres et al. (2010) 40 in which smoking, obesity and a large WC were associated with a negative health self-assessment.

There are limitations to this study associated with the cross-sectional model, which may interfere with causal evaluations; however, there was sufficient information to observe associations and ORs for the data. The euglycaemic clamp method is considered the most reliable method for assessing IR; however, because it is expensive and invasive, we used the HOMA-IR as a simple, minimally invasive and inexpensive method that has been demonstrated as a useful tool for epidemiological studies 7,21.

The main strength of this study was the identification of the prevalence of IR in bank clerks, who share similar challenges and occupational hazards with millions of other workers because the service sector employs approximately 70% of the country's workforce. Epidemiological studies involving insulin dosing and HOMA-IR calculation are rare and costly; therefore, such studies may advance knowledge in this area.

Our results show that the prevalence of IR among bank employees is high and that IR is associated with MS. CVD- and MS-associated metabolic abnormalities were also observed, which suggests that IR may serve as a risk factor in this group of professionals because IR precedes the development of type 2 DM and other metabolic changes. Considering the overall health status of these workers, our findings support the need to improve health promotion in banking workplaces, and the results may offer a significant contribution to occupational health.

## AUTHOR CONTRIBUTIONS

Salaroli LB participated in the study and product design, data acquisition, analysis and interpretation of the data, and drafting and revising of the manuscript. Cattafesta M participated in the article design, data acquisition, analysis and interpretation of the data, and drafting and revising of the manuscript. Molina MC participated in the data acquisition, data interpretation, and drafting and revising of the manuscript. Zandonade E participated in the analysis and interpretation of the data and drafting and revising of the manuscript. Bissoli NS participated in the study and product design, data acquisition, analysis and interpretation of the data, and drafting and revising of the manuscript.

## Figures and Tables

**Table 1:** Characteristics related to work, self-perceived health status, anthropometric and haemodynamic profile and prevalence of insulin resistance in the studied sample, Vitória/ES-Brazil.

		HOMA-IR		
Variables	TOTAL N (%)	High N (%)	Normal N (%)	*p* value
**Worked hours**				0.747
≤ 6 hours	199 (40)	19 (36.5)	180 (40.4)	
8 hours	268 (53.8)	29 (55.8)	239 (53.6)	
> 8 hours	31 (6.2)	4 (7.7)	27 (6.1)	
**Interval**				0.416
< 1 hour	196 (39.8)	18 (34.6)	178 (40.5)	
≥ 1 hour	296 (60.2)	34 (65.4)	262 (59.5)	
**Time at the bank**				0.604
≤ 5 years	129 (26.1)	12 (23.1)	117 (26.4)	
> 5 years	366 (73.9)	40 (76.9)	326 (73.6)	
**Office**				0.570
General direction	277 (55.6)	27 (51.9)	250 (56.1)	
Agencies	221 (44.4)	25 (48.1)	196 (43.9)	
**Self-perceived health**				0.028
Very good	122 (24.5)	10 (19.2)	112 (25.2)	
Good	292 (58.8)	29 (55.8)	263 (59.1)	
Regular	70 (14.1)	8 (15.4)	62 (13.9)	
Poor	13 (2.6)	5 (9.6)	8 (1.8)	
**Physical activity**				0.893
Active	138 (27.7)	14 (26.9)	124 (27.8)	
Sedentary	360 (72.3)	38 (73.1)	322 (72.2)	
**BMI (kg/m^2^)**				0.001
Low weight	8 (1.6)	0 (0.0)	8 (1.8)	
Eutrophic	223 (44.8)	3 (5.8)	220 (49.3)	
Overweight	181 (36.3)	20 (38.5)	161 (36.1)	
Obesity	86 (17.3)	29 (55.8)	57 (12.8)	
**WC (cm)**				0.001
Normal	241 (48.5)	5 (9.6)	236 (53.0)	
High	256 (51.5)	47 (90.4)	209 (47)	
**BP (mmHg)**				0.001
Amended	132 (26.5)	25 (48.1)	107 (27)	
Normal	366 (73.5)	27 (51.9)	339 (76)	

N = 498, different values from the mean data loss

Chi-square test

HOMA-IR: Homeostasis model assessment; BMI: body mass index; WC: waist circumference; and BP: blood pressure.

**Table 2:** Biochemical, anthropometric, and haemodynamic indicators in individuals with and without insulin resistance, Vitória/ES-Brazil.

	HOMA-IR										
	Normal* N = 446					High** N = 52					
Variables	Mean	SD	Median	P25	P75	Mean	SD	Median	P25	P75	*p* value
**us-CRP (mg/dL)**	2.82	4.91	1.50	0.67	3.22	4.47	5.25	2.71	1.25	6.35	0.010
**Fasting plasma glucose (mg/dL)**	85.60	14.43	84.00	78.00	90.00	110.56	46.71	93.50	88.00	107.50	0.001
**Fasting insulin (mcUI/ml)**	4.86	2.79	4.10	2.60	6.50	17.73	7.63	15.85	14.00	19.75	0.001
**Total cholesterol (mg/dL)**	190.30	35.99	189.00	165.00	212.00	206.08	41.76	213.00	172.00	234.00	0.012***
**HDL-c (mg/dL)**	49.62	13.35	46.00	39.20	58.00	43.48	10.33	41.50	36.80	47.50	0.002
**LDL-c (mg/dL)**	120.09	54.56	116.95	94.40	138.60	124.14	36.20	124.45	93.90	153.10	0.200
**VLDL-c (mg/dL)**	22.64	12.01	19.80	15.00	27.60	35.22	17.80	30.10	24.20	43.60	0.001
**Triglycerides (mg/dL)**	117.70	81.02	99.00	74.00	140.00	195.52	131.33	153.50	122.00	226.00	0.001
**Uric acid (mg/dl)**	5.57	3.65	5.20	4.20	6.40	6.32	1.78	6.20	4.90	7.40	0.001
**Weight (kg)**	71.47	15.17	70.00	59.80	80.60	87.78	13.89	87.55	79.60	96.55	0.001
**BMI (kg/m^2^)**	25.24	4.16	24.70	22.30	27.53	31.47	5.02	31.05	28.14	35.25	0.001
**WC (cm)**	87.04	12.38	87.00	78.00	95.00	102.13	11.29	102.50	95.50	110.00	0.001***
**SBP (mmHg)**	125.14	17.09	124.00	113.00	136.00	136.97	19.92	137.25	122.00	149.00	0.001***
**DBP (mmHg)**	79.34	11.39	78.50	71.00	86.00	85.90	11.59	84.50	77.25	92.75	0.001***

Values are given as the mean and SD (standard deviation), median, and 25^th^ and 75^th^ percentiles.

∗ N = 446

∗∗ N = 52

Mann-Whitney test

∗∗∗ Student's t-test

HOMA-IR: Homeostasis Model Assessment; us-CRP: C-reactive protein ultrasensitive; HDL-c: high-density lipoprotein; LDL-c: low-density lipoprotein; VLDL-C: very-low-density lipoprotein; BMI: body mass index; WC: waist circumference; SBP: systolic blood pressure; and DBP: diastolic blood pressure.

**Table 3:** Regression results, gross and adjusted for insulin resistance, Vitória/ES-Brazil.

	Statistical analysis					
	Chi-square			Multivariate analysis		
Variables	*p* value	OR	CI 95%	P value (β beta)	adjusted OR	CI 95%
**Physical activity**	0.893			0.879		
Active		1			1	
Sedentary		1.04	0.548-1.996		1.06	0.502-2.240
**Worked hours**	0.747			0.389		
≤ 6 hours		1	1		1	1
8 hours		0.87	0.473-1.601	0.177	0.28	0.047-1.758
> 8 hours		0.71	0.225-2.254	0.367	0.37	0.044-3.159
**Time at the bank**	0.604			0.220		
≤ 5 years		1			1	
> 5 years		0.84	0.424-1.648		0.60	0.268-1.354
**Office**	0.570			0.985		
General direction		1			1	
Agencies		1.18	0.664-2.099		1.00	0.510-1.986
**Interval**	0.416			0.124		
<1 hour		1			1	
≥ 1 hour		1.28	0.703-2.344		4.11	0.680-24.836
**How do you consider your own health?**	0.028			0.276		
Very good		1			1	
Good		1.23	0.582-2.620		0.72	0.303-1.705
Regular		1.44	0.542-3.851		0.49	0.161-1.509
Poor		7.00	1.925-25.458		2.03	0.447-9.216
**BMI (kg/m^2^)**	0.001			0.000		
Eutrophic		1			1	
Overweight		8.28	2.418-28.365	0.018	4.97	1.314-18.830
Obesity		33.92	9.968-115.415	0.000	17.87	4.362-73.212
**WC (cm)**	0.001			0.044		
Normal		1			1	
High		10.61	4.144-27.189		3.28	1.035-10.381
**BP (mmHg)**	0.001			0.075		
Normal		1			1	
Amended		2.93	1.633-5.270		1.84	0.941-3.614

ORs were adjusted to the other variables using logistic regression with 95%CIs for the presence of IR based on the HOMA index.

HOMA-IR: Homeostasis Model Assessment; OR: odds ratio; CI: confidence interval; BMI: body mass index; WC: waist circumference; and BP: blood pressure.
